# Microenvironment and Radiation Therapy

**DOI:** 10.1155/2013/685308

**Published:** 2012-12-04

**Authors:** Michio Yoshimura, Satoshi Itasaka, Hiroshi Harada, Masahiro Hiraoka

**Affiliations:** ^1^Department of Radiation Oncology and Image-applied Therapy, Graduate School of Medicine, Kyoto University, 54 Shogoin Kawahara-cho, Sakyo-ku, Kyoto 606-8507, Japan; ^2^Group of Radiation and Tumor Biology, Career-Path Promotion Unit for Young Life Scientists, Kyoto University, 54 Shogoin Kawahara-cho, Sakyo-ku, Kyoto 606-8507, Japan

## Abstract

Dependency on tumor oxygenation is one of the major features of radiation therapy and this has led many radiation biologists and oncologists to focus on tumor hypoxia. The first approach to overcome tumor hypoxia was to improve tumor oxygenation by increasing oxygen delivery and a subsequent approach was the use of radiosensitizers in combination with radiation therapy. Clinical use of some of these approaches was promising, but they are not widely used due to several limitations. Hypoxia-inducible factor 1 (HIF-1) is a transcription factor that is activated by hypoxia and induces the expression of various genes related to the adaptation of cellular metabolism to hypoxia, invasion and metastasis of cancer cells and angiogenesis, and so forth. HIF-1 is a potent target to enhance the therapeutic effects of radiation therapy. Another approach is antiangiogenic therapy. The combination with radiation therapy is promising, but several factors including surrogate markers, timing and duration, and so forth have to be optimized before introducing it into clinics. In this review, we examined how the tumor microenvironment influences the effects of radiation and how we can enhance the antitumor effects of radiation therapy by modifying the tumor microenvironment.

## 1. Introduction

How radiation therapy shows antitumor effects is important in understanding the relationship between the microenvironment and radiation therapy. Cytotoxicity due to radiation is primarily attributed to damage to genomic DNA which contains all the genetic instructions for the development and functions of all living organisms. Radiation can affect atoms and/or molecules in the cells (such as water) and produce free radicals. Because free radicals are highly reactive, they damage genomic DNA, resulting in cell death. This is a so-called indirect action of radiation. On the other hand, when radiation is directly absorbed by DNA, the atoms in the DNA are ionized and damaged. This is a so-called direct action of radiation. Whether radiation acts directly or indirectly depends on the linear energy transfer (LET) of radiation, which is the energy transferred per unit length of track. The direct action is dominant with heavy charged ion beams whose LETs are high. Meanwhile, about two thirds of the biological damage due to X-, *γ*-rays, and proton beams is caused by indirect action because their LETs are low. Thus, except for heavy charged ion beams, damage to genomic DNA is mainly caused by the indirect effects of free radicals and becomes permanent in the presence of oxygen. Therefore, tumor hypoxia and angiogenesis, which influence tumor hypoxia, have been extensively studied in order to improve the antitumor effects of radiation therapy.

## 2. Tumor Microenvironments That Affect the Therapeutic Effect of Radiation Therapy

The microenvironment of malignant solid tumors is totally different from that of normal tissues, being characterized by marked diversities in pH, the distribution of nutrients, and oxygen concentrations, and so forth [1–4]. To understand this heterogeneity is important in cancer radiation therapy because it influences the effect of ionizing radiation through various mechanisms as described in the following. Since the tumor microenvironment is a unique feature, it can be a potent target for cancer therapy. However, the tumor microenvironment is not stable and is changed by treatments, so we have to consider effects on the microenvironment due to both radiation therapy and tumor microenvironment-targeting treatments that can influence the therapeutic outcome.

### 2.1. Tumor Hypoxia

#### 2.1.1. Tumor Hypoxia and Radioresistance

In 1955, Thomlinson and Gray reported a milestone study showing that partial oxygen pressure (pO_2_) is highly diverse in a malignant solid tumors; some regions are well oxygenated and others are exposed to low oxygen conditions, that is, hypoxia [3]. It has been reported that the hypoxic fraction (pO_2_ < or = 2.5 mm Hg) is approximately 25% in malignant tumors such as uterine cervix cancers, head and neck cancers, and breast cancers [5]. In contrast, there is no region where pO_2_ values are lower than 12.5 mm Hg in normal tissues such as normal breast tissues [6]. Tumor hypoxia has drawn considerable attention in radiation oncology because it has been strongly associated with radioresistance of malignant tumors, tumor recurrence after radiation therapy, and poor prognosis of cancer patients after radiation therapy, and so forth [7–9]. 

#### 2.1.2. Chronic and Acute Hypoxia

Tumor hypoxia can be grouped into two distinct categories; chronic hypoxia and acute hypoxia, according to the causative factors and the duration for which tumor cells are exposed to hypoxic conditions [7, 10]. 

**Figure 1:**
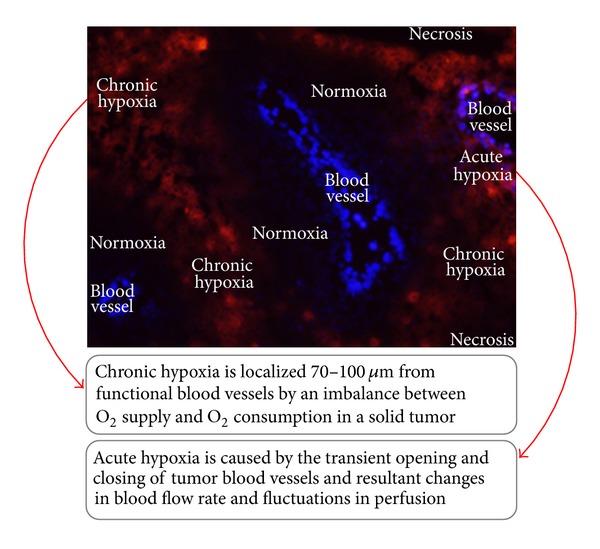
Chronic and acute hypoxia. See main text for details (Modified figure from [14]).

Cancer cells generally have unique characteristics, such as accelerated proliferative signaling, evasion of growth suppressors, replicative immortality, and deregulated cellular energetics [11]. Also, vasculatures in malignant tumors are different from those in normal tissues and are functionally and structurally defective in most malignant solid tumors [12]. These peculiarities are known to cause an imbalance between oxygen supply and oxygen consumption in malignant solid tumors and to be major causative factors in severely compromised oxygenation in some parts of malignant tumors [1–4]. Proliferation of tumor cells is dependent on the supply of oxygen and nutrients; therefore, a tumor blood vessel is surrounded by actively proliferating cancer cells. This is generally called a normoxic region [2, 3, 13]. On the other hand, cancer cells inevitably die in areas approximately 100 *μ*m from tumor blood vessels, known as necrotic regions [2, 3, 13]. Between these two distinct regions, there are chronically hypoxic regions in which cancer cells obtain minimal levels of oxygen molecules from tumor blood vessels, adequate for their survival but insufficient for their active proliferation (Figure 1) [2, 3, 13]. Thus, most malignant tumors individually grow as a conglomerate of so-called microtumor cords [2, 3, 13].

Acute hypoxia was first recognized by Brown et al. in 1979 [10]. They reported that structurally and functionally anomalous tumor vasculatures cause the transient opening and closing of blood vessels. This leads to changes in the blood flow rate and fluctuations in perfusion and ultimately causes the generation of transient hypoxia even within 70 *μ*m of tumor blood vessels (Figure 1). It is said that at least 20% of cancer cells experience acute hypoxia in malignant solid tumors. Both acute/intermittent/cycling and chronic hypoxia have received much attention because of their relevance to the malignancy and radioresistance of cancer cells [15, 16].

#### 2.1.3. Mechanism behind Radioresistance of Cancer Cells under Hypoxia

Extensive research in the field of radiation biology and radiation oncology has revealed that cancer cells become approximately 2-3 times more radioresistant under hypoxic conditions than under normoxic conditions. This phenomenon is known as the oxygen effect. The mechanism behind the oxygen effect has not yet been fully elucidated. However, it is widely believed that oxygen acts at the level of the generation of free radicals [7, 13, 17]. Ionizing radiation literally induces ionization of target genomic DNA or intracellular molecules such as water, and produces highly reactive radicals. Under oxygen-available conditions, molecular oxygen oxidizes the DNA radicals, leading to the formation of irreparable DNA damage. On the other hand, under hypoxic conditions, oxygen-depletion is known to primarily disturb the production of reactive and cytotoxic species due to ionizing radiation. Moreover, DNA radicals, which are barely produced under hypoxia, can be chemically reduced by sulfhydryl (SH) group-containing materials, resulting in the prevention of DNA damage. Thus, irreparable DNA double strand breaks (DSBs) are significantly less serious in the absence of oxygen, leading to hypoxia-related radioresistance of cells.

### 2.2. HIF-1

In addition to radiochemical mechanisms, hypoxia is also known to increase tumor radioresistance at the tissue level through some biological mechanisms. Accumulated evidence revealed the important role of a transcription factor, hypoxia-inducible factor 1 (HIF-1) [1, 14, 18–20]. 

#### 2.2.1. Regulation of HIF-1 Activity

**Figure 2:**
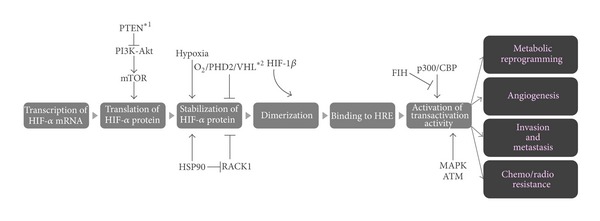
Regulation of HIF-1 activity. See main text for details.

HIF-1 is a heterodimeric factor composed of an *α*-subunit (HIF-1*α*) and a *β*-subunit (HIF-1*β*). Its hypoxia-dependent activity is regulated at multiple levels, such as translational initiation, degradation/stabilization, and upregulation of transactivation activity of HIF-1*α* (Figure 2). In the presence of oxygen, HIF-1*α* is hydroxylated by prolyl hydroxylases (PHDs) and subsequently ubiquitinated by a pVHL-containing E3 ubiquitin ligase, resulting in rapid degradation [21–24]. On the other hand, HIF-1*α* is stabilized under hypoxic conditions because of a decrease in PHD activity and interacts with HIF-1*β*. The resultant HIF-1 binds to its cognate transcriptional enhancer sequence, the hypoxia-responsive element (HRE), and induces the expression of various genes related to the adaptation of cellular metabolism to hypoxia (the switch from oxidative to anoxic respiration) [25], escaping from hypoxia (invasion and metastasis of cancer cells) [26, 27], and reduces hypoxia (angiogenesis) [28, 29], and so forth.

In addition to the PHDs-VHL-mediated mechanism, other mechanisms have been reported to function in the regulation of HIF-1 activity (Figure 2). For example, stability of HIF-1*α* is also regulated in a receptor of activated protein kinase C (RACK1)-dependent manner [30]. Interaction with RACK1 leads to the oxygen-independent degradation of HIF-1*α* because RACK1 competitively inhibits the interaction of HIF-1*α* to heat shock protein 90 (HSP90) which stabilizes the HIF-1*α* protein. Also, it was recently elucidated that HIF-1*α* protein synthesis depends on a phosphatidylinositol 3-kinase (PI3 K-) Akt-mammalian target of the rapamycin (mTOR) signaling transduction pathway because of the existence of a polypyrimidine tract in the 5′-untranslated region of HIF-1*α* mRNA [31, 32]. Furthermore, the post-translational modification of HIF-1*α* also plays a critical role in stimulating the transactivational activity of HIF-1 [33]. Under normoxic conditions, factor inhibiting HIF-1 (FIH-1) becomes active and hydroxylates an asparagine residue (N803) of HIF-1*α* [21, 33]. The hydroxylation blocks the recruitment of co-factors p300 and CBP, resulting in the suppression of HIF-1^,^s transactivational activity. Phosphorylation of HIF-1*α* by mitogen-activated protein kinase (MAP kinase) and ERK signaling pathways is also known to play an important role in the upregulation of its transactivation activity.

#### 2.2.2. Radioresistance of Tumor Cells via a HIF-1-Mediated Biological Mechanism

An interesting model for the role of HIF-1 in tumor radioresistance was proposed recently; (1) radiation activates HIF-1 in a solid tumor as a result of both the increase in oxidative stress [18, 19] and improvement in glucose and oxygen availabilities [1, 14, 34, 35], (2) HIF-1 induces the expression of VEGF, (3) VEGF protects endothelial cells from the cytotoxic effects of radiation, and (4) the radioprotected tumor blood vessels assure the supply of oxygen and nutrients to tumor cells and promote tumor growth [18, 35–37]. The feasibility of this model has been confirmed by the following data. Optical imaging using an HIF-1-dependent reporter gene revealed that intratumor HIF-1 activity is dramatically induced by radiation therapy [18, 34, 35, 38, 39]. A hypoxia-conditioned medium, which contained a high level of VEGF, significantly reduced the incidence of radiation-induced apoptosis of human umbilical vein endothelial cells *in vitro* [35–37]. An HIF-1 inhibitor, YC-1, or a neutralizing antibody against VEGF dramatically induced apoptosis of endothelial cells and reduced microvessel density after radiation therapy, resulting in a radiosensitizing effect in a tumor growth delay assay [18, 35, 40].

In addition to such indirect mechanisms of action, our group recently revealed a direct function of HIF-1 in tumor recurrence after radiation therapy [41]. We first developed a sophisticated strategy to track the post-irradiation fate of the cells which were present in perinecrotic regions at the time of radiation. The cell tracking experiment revealed that the perinecrotic cells predominantly survived radiation therapy and directly caused recurrent tumors. Although the perinecrotic cells did not originally express HIF-1, they acquired HIF-1 activity after surviving radiation. Interestingly, the activation of HIF-1 triggered the migration of the radiosurviving cells towards functional tumor blood vessels and eventually caused tumor recurrence. 

### 2.3. Tumor Angiogenesis

 For solid tumors, angiogenesis is necessary to grow over a diameter of 2 mm to obtain oxygen and nutrients. The angiogenic switch is a critical step in the process of tumor growth; an initial avascular tumor nodule becomes a rapidly growing, highly vascularized tumor. The concept that blockage of angiogenesis could be a target in cancer therapy was proposed in 1971 by Judah Folkman [42]. Antiangiogenic therapy has an advantage that targeting endothelial cells without genetic mutations should lead to less resistance to the antiangiogenic treatment. However, the use of antiangiogenic agents has a limitation in that they cannot eradicate tumors as monotherapy and need to be combined with cytotoxic therapy. The combination of antiangiogenic therapy and radiation therapy showed synergic effects in several preclinical models despite the prediction that antiangiogenic therapy would increase tumor hypoxia. In clinics, the role of the combination of antiangiogenic therapy and radiation therapy is still under investigation.

#### 2.3.1. Combination of Radiation Therapy and Antiangiogenic Therapy

The synergistic effects of the combination of radiation therapy and antiangiogenic agents have been reported in several preclinical studies (Table 1). Gorski et al. [36] showed that an anti-VEGF antibody alone did not suppress the growth of U87 glioblastomas, but when it was combined with radiation, it showed a significant improvement in terms of antitumor effects. Kozin et al. [43] observed that DC101, an anti-VEGFR2 antibody, enhanced the effects of radiation therapy in 54A non-small cell lung cancer and U87. Several tyrosine kinase inhibitors were developed to block the VEGF receptor and other receptors that are proangiogenic. For example, Huber et al. [44] reported that SU11657, which inhibits VEGF, PDGF and C-kit, also enhanced the effects of radiation with chemotherapy on A431 tumors, and that triple inhibition was more effective than blockade of each single target. Synergistic antitumor effects in the combination with radiation therapy were also reported for angiostatin [45–47] and endostatin [48–50]. In contrast, Murata et al. [51] observed that the concurrent treatment of mouse breast carcinoma xenografts with TNP-470 and fractionated radiation therapy resulted in reduced tumor control and tumor oxygenation decreased. Although many preclinical studies showed enhanced antitumor effects in the combination of antiangiogenic agents and radiation therapy, this study indicated the possibility that a schedule of both radiation therapy and antiangiogenic therapy could influence the therapeutic outcome. 

#### 2.3.2. Vascular Normalization

 Tumor angiogenesis is characterized by tortuous, irregular, and immature vessels, and microvessel density is inhomogeneous in the tumors. In addition, poor coverage with pericytes leads to a marked increase in vessel leakiness and high interstitial pressure in the tumor. Therefore, blood flow in the tumor is insufficient to supply enough oxygen and nutrients even in well vascularized areas in the tumor. Jain and colleagues [12] proposed the term “vascular normalization.” At the time of angiogenic switch, proangiogenic factors are more dominant over antiangiogenic factors and provoke marked angiogenesis in tumors. If proangiogenic factors and antiangiogenic factors are balanced, disappearance of immature microvessels and an increase in pericyte coverage lead to a transient increase in blood flow and lower interstitial pressure. Winkler et al. [55] demonstrated that DC 101 (a VEGFR2 inhibitor) treatment transiently increased tumor oxygenation and synergistic effects were observed when radiation was combined during this period. This concept can also explain why the combination of antiangiogenic agents and cytotoxic chemotherapy showed improved overall survival for colorectal carcinoma. These findings raised a question about the best schedule to obtain maximal effects of combination of radiation and antiangiogenic therapy. 

#### 2.3.3. Sequence of Radiation Therapy and Antiangiogenic Therapy

If antiangiogenic agents can improve the tumor oxygenation by vascular normalization, the timing of radiation should be after antiangiogenic therapy, and preclinical studies indicated the possibility that its long-term use may lead to an increase in tumor hypoxia. Dings et al. [52] studied the combination of bevacizumab, anginex, an antiangiogenic peptide, and radiation therapy. They found significantly increased tumor oxygenation in the four days after the start of treatment. When radiation was combined during this period, tumor growth delay was extended. Although our group could not show a transient increase in tumor hypoxia with bevacizumab treatment, we could show an increase in tumor hypoxia 72 hours after administration by HIF-1 imaging [40]. If the radiation was combined 24 hours after bevacizumab treatment when HIF-1 activity was not upregulated, enhanced antitumor effects were observed; however, 72 hours after bevacizumab treatment when HIF-1 activity was upregulated, antitumor effects were lower than radiation alone. If an optimal time window for combining radiation with antiangiogenic agents exists, its duration of is estimated to be both tumor and host dependent. The development of hypoxia imaging which can monitor the changes in tumor hypoxia repeatedly is needed to determine the optimal time window in clinics. 

Not all antiangiogenic agents seem to have a vascular normalization window. Williams et al. [58] found that ZD6474, an inhibitor of VEGFR and EGFR, was most effective when it was administered 30 minutes after radiation therapy as compared to concomitant administration or radiation alone. PTK787, a VEGFR2 inhibitor, was also most effective when administered after fractionated irradiation, but not before or during radiation [56]. 

As previously described, VEGF expression induced by HIF-1 upregulation from radiation therapy can protect tumor endothelial cells from apoptosis due to radiation therapy. Both an HIF-1 inhibitor, YC-1, and a neutralizing antibody against VEGF dramatically induced apoptosis of endothelial cells and reduced microvessel density after radiation therapy and delayed tumor growth [18, 35, 40]. Endostatin also downregulated VEGF after radiation therapy and induced apoptosis, reducing proliferation of endothelial cells after radiation therapy and significantly delayed tumor growth [49]. These effects on endothelial cells are independent of vascular normalization windows and can be another factor to determine the optimal timing of the combination of antiangiogenic therapy and radiation.

#### 2.3.4. Endothelial Cells and Radiosensitivity

Garcia-Barros et al. [70] showed that apoptosis of endothelial cells is mediated by rapid generation of sphingolipid ceramide through the hydrolysis of cell membrane sphingomyelin by the acid sphingomyelinase (ASM) enzyme. In this study, a single high-dose radiation (>15 Gy) was used and would be relevant only to hypofractionated stereotactic radiotherapy such as stereotactic body radiotherapy (SBRT) or stereotactic radiosurgery (SRS). In this study, endothelial cell apoptosis was directly related to tumor radiosensitivity. High local control rates of SBRT and SRS suggest that vascular damage may play an important role in the response of SBRT or SRS in clinics. 

## 3. Targeting the Tumor Microenvironment to Improve the Effects of Radiation Therapy

In recent years it has become increasingly clear that the efficacy of radiation therapy is influenced by the tumor microenvironment. Several classes of agents which modulate microenvironmental factors have been developed, and some of them have radiosensitizing potential. The two major microenvironmental factors which influence the radiosensitivity of tumor cells are oxygenation and angiogenesis.

### 3.1. Hypoxia and Radiosensitization

Hypoxia, which is commonly seen in malignant solid tumors, is known to be one of the most important characteristics in the tumor microenvironment and is associated with tumor radioresistance. Since the 1950s, many scientists have proposed a hypoxic environment to make tumor cells more radioresistant compared with a well-oxygenated tumor environment. 

To overcome hypoxia-related radioresistance, several methods to increase oxygen delivery, radiosensitizers for hypoxic tumor cells, hypoxic cytotoxins, and HIF-1 inhibitors have been developed (Table 2). 

#### 3.1.1. Increase in Oxygen Delivery

Several groups have tried to increase the delivery of oxygen to tumor lesions through blood flow. Representative treatment methods are hyperbaric oxygen therapy, carbogen with nicotinamide, blood transfusion and erythropoietin.

(1) *Hyperbaric Oxygen Therapy*. Hyperbaric oxygen (HBO) therapy is the inhalation of 100% oxygen at elevated pressure. It is a promising approach to cope with tumor hypoxia by dissolving oxygen in the plasma and delivering it to tumor sites independent of hemoglobin while increasing the concentration of oxygen in the tumor area. The first report about HBO with radiation therapy was published in the 1960s, and since then several clinical trials have been conducted for solid tumors such as cervical cancer, head and neck cancer, bladder cancer and malignant glioma, but the benefit of this method remains controversial [71–76].

(2) *Carbogen with Nicotinamide.* Carbogen is a mixture of O_2_ and CO_2_ gas. Breathing carbogen is known to reduce diffusion-limited hypoxia. Nicotinamide, the amide derivative of vitamin B6, is a vasoactive agent which counteracts acute hypoxia; administering nicotinamide reduces perfusion-related acute hypoxia. In addition, Nicotinamide is known to inhibit Poly ADP-ribose polymerase I which is a critical enzyme in single stranded DNA break repair [77], and many studies have shown that the inhibition of poly-ADP-ribose polymerase enhances tumor radiosensitivity [78–80]. This could be also one of the rationales for the radiosensitizing effect of the combination therapy with carbogen and nicotinamide. Normobaric carbogen only or carbogen plus nicotinamide therapies have been used with radiation therapy to overcome the hypoxic radioresistance of malignant tumors. In the 1990s, a schedule of accelerated radiotherapy with carbogen and nicotinamide (ARCON) was also proposed. However, the addition of carbogen breathing to definitive RT did not appear to improve the likelihood of local control for T2-4 head and neck cancers [81]. Several clinical trials using radiotherapy with carbogen and nicotinamide including ARCON are now ongoing for head and neck cancer and bladder cancer [82, 83]. The treatment outcome and morbidity will determine the therapeutic benefit of these treatment strategies.

(3) *Hemoglobin Modification (Red Blood Cell Transfusion and Erythropoietin). *Several preclinical and clinical studies have shown that a low hemoglobin level is related to tumor hypoxia [84]. An increase in hemoglobin levels with blood cell transfusions, erythropoietin, and erythropoiesis-stimulating agents (ESAs) could be a promising method to enhance the response to radiation therapy by increasing the oxygen concentration of the tumor. The use of recombinant erythropoietin or erythropoiesis-stimulating agents (ESAs) with radiation therapy in patients with head and neck cancer has been tested. However, radiation therapy with hemoglobin modification has no impact on clinical radiation therapy [85–89].

#### 3.1.2. Nitromidazole Derivatives

Nitroimidazole-based agents such as misonidazole and nimorazole were found to mimic the effect of oxygen and enhance the cytotoxic effect of ionizing radiation on hypoxic malignant tumors. Several clinical trials using these drugs have been conducted. It was reported that the use of an effective dose of misonidazole caused late peripheral neuropathy, while nimorazole, a less toxic nitroimidazole-derivative, could be used at higher doses and significantly improved the radiotherapeutic effect of supraglottic and pharyngeal cancers [90–93]. 

#### 3.1.3. Hypoxic Cytotoxins

We can utilize hypoxia as a specific target of treatment. The most representative hypoxia-activated prodrug is tirapazamine, and its mechanism of action has already been well established [7, 94]. Tirapazamine is subjected to one-electron reduction to a radical anion. The radical anion can be reversibly oxidized to the parental compound in the presence of molecular oxygen [95], but can be further converted to a toxic hydroxyl radical or to an oxidizing radical in the absence of oxygen [96]. Both of the resultant radicals cause DNA DSBs, single-strand breaks, and base damage, resulting in cell death, especially under hypoxic conditions. Because hypoxic tumor cells are the most radiation-resistant cells in malignant solid tumors, tirapazamine and radiation act as complementary cytotoxins; namely, each one kills the cells resistant to the other, thereby enhancing the efficacy of radiation against the tumor [7]. Despite promising early results [97, 98], a phase III trial of tirapazamine in combination with radiation therapy showed no significant difference in failure-free survival, time to locoregional failure, or quality of life [99]. Currently, new improved TPZ analogues with higher hypoxic potency are being developed [100].

### 3.2. HIF-1 Inhibitors

**Figure 3:**
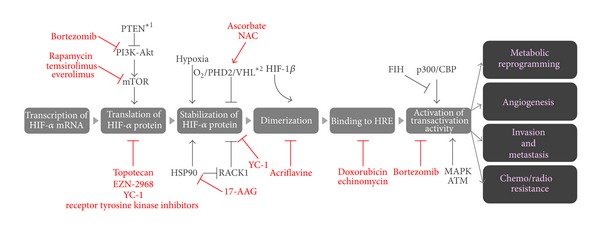
HIFs Inhibitors. See main text for details.

Basic and clinical researches have confirmed that the expression level of HIF-1*α*, as well as absolute low pO_2_, correlates with a poor prognosis and incidences of both tumor recurrence and distant tumor metastasis after radiation therapy [7–9, 101–103]. Each of the multiple steps responsible for the activation of HIF-1 has been exploited as a therapeutic target (Figure 3).

One of the major targets is the mechanism behind the stabilization of HIF-1*α* protein, because it is the most influential step in HIF-1 activity. YC-1, which was primarily synthesized with the aim of activating soluble guanylate cyclase and inhibiting platelet aggregation, was reported to suppress the expression of HIF-1 target genes through the suppression of HIF-1*α* accumulation and to increase the antitumor efficacy of radiation therapy significantly [18, 35, 104, 105]. An HSP90 inhibitor, 17-allylamino-17-demethoxygeldanamycin (17-AAG), facilitates the RACK1-dependent ubiquitination of HIF-1*α*, resulting in its degradation through proteasome. Also, antioxidant reagents such as ascorbate and *N*-acetyl cystein (NAC), promote the degradation of HIF-1*α* protein by reducing Fe^3+^ to Fe^2+^, which functions as a cofactor in the PHDs-VHL-dependent degradation of HIF-1*α* protein [106]. 

Inhibiting the dimerization of HIF-1*α* with HIF-1*β* was also targeted because it is required for HIF-1 DNA-binding and transcriptional activity. Lee et al. identified acriflavine as an inhibitor of the dimerization by directly binding to HIF-1*α* [107]. They reported that acriflavine treatment inhibited intratumoral expression of angiogenic cytokines, mobilization of angiogenic cells into peripheral blood, and tumor vascularization, resulting in the prevention and arrest of tumor growth [107]. 

Another approach is to inhibit the function of key signaling pathways which up-regulate the expression of HIF-1*α*, such as the PI3 K-Akt-mTOR and Ras signaling pathways [31, 32, 108]. An mTOR inhibitor, RAD-001, actually reduced the level of HIF-1*α* protein and its downstream gene products in a mouse model of prostate cancer with high oncogenic Akt activity [109]. Other mTOR inhibitors, such as rapamycin, temsirolimus (CCI-779), everolimus (RAD-001), also showed the same effect [110]. In addition, it was reported that doxorubicin and echinomycin suppress the function of HIF-1 by inhibiting HIF-1′s binding to HRE [107, 111]. 

Because HIF-1 directly and indirectly functions in tumor recurrence after radiation therapy as described above, HIF-1 inhibitors, as well as tirapazamine, have been confirmed to enhance the therapeutic effect of radiation [18, 35, 38, 41, 112, 113]. However, it has also been reported that the inhibition of HIF-1 with unsuitable timing suppresses rather than enhances the effect of radiation therapy because its antiangiogenic effect increases the radioresistant hypoxic fraction in malignant solid tumors [35]. Accumulated evidence indicates that the suppression of the postirradiation upregulation of HIF-1 activity is important for the best therapeutic benefit [18, 20, 35, 41].

### 3.3. Angiogenesis and Radiosensitization

Angiogenesis is essential for tumor growth because it allows tumor cells to obtain enough oxygen and nutrients for their survival; anti-angiogenesis has played a major role in cancer research. Recently, many antiangiogenic agents have been developed, and some of these are in clinical use. However, combination treatment of antiangiogenic agents and radiotherapy in clinics is still in its early stages. No antiangiogenic agents have yet been approved for clinical treatment in combination with radiation therapy.

#### 3.3.1. Angiostatin and Endostatin

Angiostatin, which is a proteolytic fragment of plasminogen and an intrinsic angiogenic inhibitor, was reported to have the potential to enhance the antitumor effects of radiation [45]. Itasaka et al. showed that endostatin, an endogenous angiogenesis inhibitor, enhanced the tumor response to radiation and blocked tumor revascularization after radiation treatment [49]. Another group reported that recombinant human endostatin radiosensitized xenografted human nasopharyngeal carcinoma in mice [50]. However, these inhibitors have not yet been clinically used in combination with radiation therapy. 

#### 3.3.2. Anti-VEGF Antibody

VEGF is one of the promising targets for anticancer therapy. Neutralization of VEGF inhibited the growth of primary tumors and metastases [53]. Blocking VEGF with a neutralizing antibody enhanced the antitumor effects of radiation in preclinical studies [36]. Another group reported that an anti-VEGF monoclonal antibody in combination with radiation led to tumor growth delay in mouse xenograft models [54]. 

Bevacizumab is a humanized monoclonal antibody which neutralizes the VEGF ligand. Bevacizumab in combination with cytotoxic chemotherapy showed a significant improvement in survival in patients with advanced colorectal or lung cancer [114, 115]. Currently bevacizumab is approved for use in combination with cytotoxic chemotherapy in those diseases. The combination therapy of bevacizumab with radiation is also a promising strategy to improve the antitumor effects. A clinical trial with a combination of radiation therapy plus 5-FU with bevacizumab followed by surgery was done and led to encouraging results in patients with locally advanced rectal cancer [116]. The combination of radiation therapy with bevacizumab resulted in promising responses in locally advanced inoperable colorectal cancer [117]. The addition of bevacizumab to neoadjuvant chemoradiotherapy using capecitabine resulted in encouraging pathologic complete response with tolerable toxicity for locally advanced rectal cancer [118]. Further clinical studies are required to assess the role of combination therapy of bevacizumab with radiation or chemoradiation in patients with rectal cancers.

A phase II study was conducted to evaluate the use of bevacizumab in combination with concurrent capecitabine and radiation therapy followed by maintenance gemcitabine and bevacizumab for patients with locally advanced pancreatic cancer. The median overall survival and the median progression-free survival time were similar to the results obtained in prior RTOG trials with conventional chemoradiotherapy [119]. This result implies that the addition of bevacizumab does not improve the efficacy of conventional chemoradiotherapy in patients with locally advanced pancreatic cancer. Currently, several clinical trials using combination therapy of bevacizumab with radiation or chemoradiation are ongoing in patients with other malignant tumors such as glioblastoma or head and neck cancers [120, 121]. 

#### 3.3.3. Anti-VEGFR Agents

DC101 is a VEGFR2 antibody, and it was reported to reduce the radiation dose necessary to control tumor models [43]. DC101 in combination with radiation showed a synergistic effect when irradiation was performed several days after the administration of DC101 [55]. Many groups have shown that the VEGF receptor tyrosine kinase inhibitors enhance the radiation response in preclinical studies. Radiation treatment with the VEGF receptor tyrosine kinase inhibitor, PTK787/ZK222584 (vatalanib), delayed tumor growth in colon tumor xenografts [57]. The combination of another VEGFR tyrosine kinase inhibitor, ZD6474(vandetanib), and radiation, led to significant enhancement of antiangiogenic, antivascular, and antitumor effects in an orthotopic model of lung cancer [59]. AZD2171 (cediranib) is a potent VEGFR tyrosine kinase inhibitor, and it has been reported to radiosensitize tumor xenografs [60, 61].Several clinical trials using these agents with radiation therapy are now being performed [122].

#### 3.3.4. Inhibitors of VEGFR2, PDGFR, c-kit and Fetal Liver Tyrosine Kinase 3

Sunitinib (SU11248) is a multityrosine kinase inhibitor of VEGFR2, PDGFR, c-kit, and fetal liver tyrosine kinase 3, and it was reported to radiosensitize tumor cells in preclinical studies [65, 66]. Now, several clinical trials using sunitinib in combination with radiation therapy are ongoing [123, 124]. 

#### 3.3.5. Other Agents

(1) *Thalidomide.* Thalidomide is an orally administered drug which inhibits angiogenesis [69] and has been recognized to have several antitumor and antimetastatic mechanisms. Radiation Therapy Oncology Group (RTOG) conducted a phase III study to compare whole-brain radiation therapy (WBRT) with WBRT combined with thalidomide for patients with brain metastases (RTOG0118), but thalidomide with radiation therapy provided no survival benefit [125].

(2) *Inhibitors of the EGFR/RAS/PI3 K/Akt/mTOR Pathway*. Preclinical studies showed that the anti-EGFR monoclonal antibody C225 (cetuximab) enhanced the radiosensitivity of tumor cells [126]. A phase III trial using a combination of cetuximab and radiation therapy significantly improved overall survival at 5 years compared with radiation therapy alone in the treatment of locally advanced head and neck squamous cell carcinoma [127]. Many other inhibitors of these pathways have been shown to enhance tumor radiosensitivity at clinically relevant doses in preclinical experiments [78, 128–132].

Qayum and colleagues showed that inhibition of EGFR- Ras-PI3 K-Akt signaling at multiple points in this pathway led to vascular normalization accompanied by improved tumor oxygenation and perfusion. Cerniglia et al. showed that erlotinib (an EGFR inhibitor) treatment of mice bearing xenografts led to reduced VEGF expression, enhanced vascular functioning in the tumors, increased blood flow, and improved oxygenation, resulting in enhancement of radiosensitivity. Moreover, Fokas and colleagues reported that a dual inhibitor of phosphoinositide-3-kinase (PI3 K) and mTOR improved vascular structure over a prolonged period. These studies have shown that inhibition of signaling through EGFR, RAS, PI3-Kinase, AKT, and mTOR results in enhanced vascular function, which may be one of the mechanisms by which inhibitors of these pathways radiosensitize tumor cells. 

## 4. Conclusion

The tumor microenvironment has been the main focus and the therapeutic target in the field of radiation biology and oncology in terms of tumor hypoxia. Understanding of the biological response to hypoxia through HIF-1 revealed many molecules and complicated pathways related to survival of cells and progression of malignancy. In addition to direct approaches to hypoxia, targeting molecular pathways related to HIF-1 pathways is promising to improve the efficacy of radiation therapy. Tumor angiogenesis is also a good target for cancer therapy. Either direct or indirect inhibition of angiogenesis can enhance the effects of radiation therapy. Since radiation therapy itself has a great impact on host cells like vascular endothelial cells, it has become clear that changes in the tumor microenvironment during therapy and the optimal timing of the combination is a key to achieving maximal therapeutic effects in the combination therapy of radiation and microenvironment targeting. However, we still have further challenges to incorporate targeting therapy for the microenvironment to improve the effects of radiation therapy in clinics, and this will lead to greater knowledge about how radiation therapy works in cancer therapy and thus further improvements in radiation therapy.

## Figures and Tables

**Table 1:** Antiangiogenic agents with radiosensitizing potentials.

Category	Representative strategies/protein/drugs	References
Endogenous angiogenesis inhibitor	Angiostatin,	[45–47]
	Endostatin	[48–50]

Anti-VEGF antibody	Bevacizumab	[36, 40, 52–54]

Anti-VEGFR antibody	DC101	[43, 55]

Anti-VEGFR tyrosine kinase inhibitor and multitarget tyrosine kinase inhibitor	Vatalanib (PTK787),	[56, 57]
	Vandetanib (ZD6474),	[58, 59]
	Cediranib (AZD2171)	[60, 61]
	Semaxanib (SU5416),	[62, 63]
	SU6668	[64]
	SU11657	[44]
	Sunitinib (SU11248)	[65, 66]

Others	TNP-470	[51, 67, 68]
	Thalidomide	[69]

**Table 2:** Strategies to overcome radioresistance of hypoxic tumor cells.

Strategies	Mechanisms/representative strategies or drugs
Hyperbaric oxygen therapy	Direct oxygen delivery to hypoxic regions
Carbogen with nicotinamide	Direct oxygen delivery to hypoxic regions/ARCON
Hemoglobin modification	Direct oxygen delivery to hypoxic regions
Nitroimidazole derivatives	Radiosensitization by mimicking the effect of oxygen/misonidazole
Hypoxic cytotoxins	Cell killing by hydroxyl radicals or an oxidizing radicals/tirapazamine
HIF-1 Inhibitors	Suppression of radioresistant phenotype of hypoxic tumor cells/YC-1

Modified figure from [14].
